# Feeling insignificant, hopeless, and unkind to oneself: lessons learned from lonely young adults during pandemic times

**DOI:** 10.3389/fpsyg.2026.1909788

**Published:** 2026-07-31

**Authors:** Alison L. Rose, Joel O. Goldberg, Gordon L. Flett, Sarah E. McComb, Taryn Nepon

**Affiliations:** 1 Department of Psychology, York University, Toronto, ON, Canada; 2 Department of Psychology, University of Toronto, Toronto, ON, Canada

**Keywords:** anti-mattering, anxiety, hopelessness, loneliness, mattering, pandemic, self-compassion

## Abstract

The current study investigated the construct of anti-mattering (feeling insignificant and invisible) and its links with loneliness and anxiety during times of pandemic social restrictions and isolation. Importantly, it also examined how levels of anti-mattering relate to levels of domain-specific measures of social hopelessness and social self-compassion. We assessed 282 undergraduate students during the initial stages of the COVID-19 pandemic. Participants completed the Anti-Mattering Scale (AMS), the UCLA Loneliness Scale, pandemic-specific measures of anxiety and worry, and measures of social self-compassion, hope, hopelessness, and social hopelessness. Anti-mattering scores were associated with lower levels of social self-compassion and hope and higher levels of hopelessness and social hopelessness. Regression analyses showed loneliness was predicted by anti-mattering, social hopelessness, and reduced hope. The findings suggest that young people who feel both lonely and insignificant are particularly vulnerable to failing to see a better tomorrow. Furthermore, results suggest when people feel their voices are unheard, they also have a tendency to harden themselves against compassion. The findings are poignantly relevant to discussions of alienation and aspects of loneliness when one's future seems bleak. Potential clinical and community health implications are discussed regarding the relevance of alleviating distressing feelings of personal insignificance beyond the period of pandemic times.

## Introduction

Lessons learned from the COVID-19 pandemic remain relevant today since this period provided, perhaps sadly, a living laboratory test of our capacity for adjustment and adaptability. It was a time of prolonged stress accompanied by uncertainty and safety concerns. This seems to have been most poignantly true with the links between pandemic isolation and elevated levels of anxiety and distress contributing to feelings of demoralization due to setbacks and the loss of typical routines (Atlas et al., 2025). Indeed, there had already been growing concerns expressed about the world-wide increasing prevalence of mental health problems in college and university students, which existed prior to the pandemic, according to the World Health Organization reports and meta-analyses findings (see Auerbach et al., 2018; Sheldon et al., 2021). Perhaps most pertinent is an observation from an esteemed developmental psychologist (Larson, 1990) who noted that periods of solitude, whether brief moments or extended seclusion, have a range of functions and meanings in the human life cycle. As such, the current study considers that we may be able to come to learn broader understandings of loneliness and mental health concerns in the life cycle of these emerging adults through investigation during this exceptional particular period of extended seclusion.

A key concept that emerged to prominence in the research literature amidst the world-wide pandemic time remains especially relevant today—specifically, a construct which focuses on individual differences in the feelings of not mattering to other people, termed anti-mattering.

The initial conceptualization of mattering by Rosenberg and McCullough (1981) focused on positive feelings of mattering to other people as reflected by such indicators as getting the attention of other people and becoming significant when people come to depend on an individual person. However, this work was followed by an extended analysis by Schlossberg (1989) who contrasted the happiness and joy that comes from feelings of mattering to others with the abject negative feelings experienced by people subjected to being marginalized and made to feel insignificant. Flett et al. (2022) operationalized this construct through developing a five-item Anti-Mattering Scale to measure the feelings that arise when people are made to feel insignificant, invisible, and feeling as though they have a voice that is not being heard by other people. The premise behind the research conducted with the Anti-Mattering Scale is that it taps a negative orientation that is not simply the opposite of positive feelings of mattering. Specifically, it has been posited that the motivational and cognitive elements that accompany a focus on perceptions of not mattering to others are quite different than the motivational and cognitive elements of positive feelings of mattering to others. Indeed, while “mattering” is about truly feeling valued by others, “anti-mattering” is more closely associated with alienation and feeling devalued or unvalued. In this regard, an investigation of social and interpersonal relatedness among university students found that elevated levels of anti-mattering were associated with reduced closeness and more disconnection from others (Nepon and Flett, 2024). Further, a meta-analysis found that anti-mattering showed a stronger association with depressive symptoms compared with general mattering (Tonini et al., 2025). Anti-mattering has been associated with higher levels of mental health problems in a sample of United Kingdom university students (Smith and McLellan, 2024) while a longitudinal study of Chinese high school students identified that over time, spikes in anti-mattering above personal baselines predicted increased loneliness (Li et al., 2026). However, research remains sparse on anti-mattering and much remains to be learned about the anti-mattering construct and how it relates to mental health outcomes.

The current study addressed two main objectives. First, our primary aim was to examine how elevated levels of anti-mattering related to levels of loneliness and anxiety experienced by emerging adults during the pandemic. Past research conducted on mattering and loneliness suggests that there is a strong negative association between mattering and loneliness (see Flett et al., 2016). The emphasis on loneliness is in keeping with concerns about the impact of physical isolation and social isolation during the pandemic on vulnerable people. Loneliness, unlike solitude, is painful, unwanted and inevitably causes suffering (Rokach, 2015). Indeed, loneliness has been associated with serious public health consequences (Hawkley and Cacioppo, 2010). Young adults were considered at particularly high risk for loneliness and mental health concerns amidst pandemic restrictions and isolation with these mental health impacts hypothesized as being due broadly to disruptions in their social lives (Lee et al., 2020).

Most relevant to the topic of personal and societal alienation is the second focus of the current study. Specifically, we sought to examine anti-mattering as it relates to social self-compassion and how feelings of not mattering relate to outcome-expectancy measures. Self-compassion is a positive psychology construct proposed by Neff (2003). Extensive work on individual differences in self-compassion has confirmed that a lack of self-compassion is implicated in poorer psychological adjustment (Neff et al., 2007a,b; MacBeth and Gumley, 2012). Self-compassion should have been a particularly key resource during the pandemic because of the need to not only care for oneself but also to recognize the shared human suffering (Schnepper et al., 2020). We examined anti-mattering and self-compassion in the current study, but with a new questionnaire that examines domain-specific social self-compassion (see Rose and Kocovski, 2021). The measure evaluates the extent to which someone manages to feel kind, caring, and not hardened despite difficult times. Social self-compassion has been shown to be associated with lower levels of shame and fewer maladaptive self-regulation strategies when feeling a sense of shame (Lear et al., 2026). Accordingly, we hypothesized in the current study that elevated levels of anti-mattering would be associated negatively with social self-compassion; individuals who are lacking in social self-compassion may be at particular risk to feeling invisible and insignificant (anti-mattering), a risk that may be even more heightened by the high isolation experienced during pandemic times. Regarding a hypothesized association with social self-compassion, initial research has focused on mattering as a positive construct and has not considered levels of anti-mattering. Past studies have confirmed that people with higher levels of mattering also tend to have higher self-compassion (Joeng and Turner, 2015; Raque-Bogdan et al., 2011). In the initial scale construction and validation study with university students, Rose and Kocovski (2021) found that elevated general mattering scale scores were associated with social self-compassion (*r* = 0.50) as well as overall life satisfaction. To our knowledge, the current study is the first research to specifically examine anti-mattering and social self-compassion.

Research on mattering and hopelessness is in its early stages and there is little investigation thus far of the association between the mattering and hopelessness constructs. However, intriguing research examining online social media posts yielded strong evidence of the presence of anti-mattering themes, significantly reflecting a sense of hopelessness (see Deas et al., 2023). Some initial research that employed self-report questionnaires has suggested links of mattering with hope. Castro Baker et al. (2021) found that the reliance facet from the Mattering Index was associated with greater levels of hope. Somers et al. (2022) also found among 206 adolescents that hope as assessed with the Children's Hope Scale (see Snyder et al., 1997) was associated with feelings of mattering in terms of brief measures of mattering in general *(r* = 0.45) and mattering by giving value to others (*r* = 0.47).

It is conceivable, if not likely, that mattering and anti-mattering experiences contribute to a future-focused self-schema centered around interpersonal expectancies. People rich in feelings of mattering to others likely hold hopeful expectations that the future will work out reasonably well for them, especially in the interpersonal domain. In contrast, however, anti-mattering ought to contribute to a view of the future that is framed in feelings of hopelessness, especially in terms of anticipated troubled or absent exchanges involving other people. Linked to loneliness, Rokach (2004) proposed that feelings of hopelessness are often part of what he considered to be a “vicious cycle” of loneliness, which may become self-perpetuating with the hopeless anticipation of continuing to be lonely into the future. Operationally, to examine this formulation, we considered that it requires the use of an interpersonal domain-specific measure, termed social hopelessness (Flett et al., 2003). Social hopelessness has been found to be linked to suicidal ideation in university students (Heisel et al., 2003) but has not been studied to date with regard to loneliness and anti-mattering. Perhaps sadly, the pandemic period provided a naturalistic and especially relevant time for examining future outlooks and social hopelessness among young people who were experiencing extensive and ongoing social isolation due to lockdowns and social distancing rules (Van de Velde et al., 2025). It was a period when the enforced social distancing and social isolation was associated with worry and anxiety conditions not only among the elderly (Flett and Heisel, 2021) but also in young people (Al-Otaibi et al., 2025).

Taken together, the current study examined the extent to which anti-mattering was associated with loneliness and anxiety in emerging adults during the early period of pandemic seclusion and further, to examine the associations of loneliness with hopelessness and troubled self-compassion. Notably, we used domain-specific social aspects of these constructs which are hypothesized to be most relevant to evaluation of interpersonal impacts.

## Methods

### Participants

The sample comprised 282 university students (219 women, 62 men, and 1 non-binary participant). Their mean age was 22.3 years (*SD* = 4.7). The sample was diverse in terms of self-reported ethnicity, with 17.7% Caucasian, 29.4% Asian/Pacific Islander, 17% Middle Eastern, 7.1% African American, 5% Latino/Hispanic, and 23.4% who reported “other.” Before the final sample (*N* = 282) participants were obtained, we removed participants who had missing data across a number of measures as well as anyone who had failed three or more “attention checks” (i.e., a self-report question inserted in between questionnaires that participants responded to, as an indicator of careful and thoughtful responding).

### Procedure

Participants were recruited through the undergraduate research participant pool at a large Canadian university. Data collection occurred in June, 2020 when the COVID-19 pandemic had resulted in most classes being only offered online; emergency quarantine and public social distancing rules restricted social gatherings and face to face interactions with laws that had been in place since March 2020. Participants received credit toward their final introductory psychology grades in exchange for their participation. We computed total scores for all of the questionnaires mentioned below and utilized the IBM SPSS software for all statistical analyses aside from the Confirmatory Factor Analysis (CFA) in which the IBM SPSS Amos software was employed. After providing their informed consent, the following self-report questionnaires were administered in an online study:

#### Anti-Mattering Scale (AMS)

This five-item scale (Flett et al., 2022) measures the degree to which people feel as though they do not matter to others. Sample items include, “How much do you feel like you don't matter?” and “To what extent have you been made to feel like you are invisible?” Items are rated on a scale ranging from 1 (*not at all*) to 4 (*a lot*), with higher scores indicating higher anti-mattering. Flett et al. (2022) have demonstrated across several studies that the AMS is a unidimensional measure with sufficient reliability and validity. Other evidence indicated that anti-mattering is a unique construct that is related to, but distinct from, feelings of mattering (Flett et al., 2022; Flett, 2022).

#### Adult Hope Scale (AHS)

This 12-item scale (Snyder et al., 1991) assesses individual differences in perceived hope using two subscales: agency (e.g., “I meet the goals that I set for myself”) and pathway (e.g., “I can think of many ways to get the things in life that are important to me”). The subscales have four items each with four filler items. We used the total hope score in this study. Extensive research attests to the reliability and validity of this scale (Snyder et al., 1991).

#### Beck Hopelessness Scale (BHS)

This 20-item scale (Beck et al., 1974) measures levels of general hopelessness for the future. Sample items include, “I might as well give up because I can't make things better for myself” and “My future seems dark to me.” The response format is true or false, with greater scores reflecting greater hopelessness. The BHS possesses good psychometric properties (Beck et al., 1974, 1990).

#### Social Hopelessness Questionnaire (SHQ)

This 20-item questionnaire (Flett et al., 2003) assesses negative beliefs in outcome expectancies in the interpersonal domain. Sample items include, “I will always have a hard time coping with some people” and “Some people do little to inspire hope in me.” Items are rated on a scale ranging from 1 (*Strongly Disagree*) to 5 (*Strongly Agree*), with greater scores indicating greater social hopelessness. Flett et al. (2003) have shown this scale is unidimensional with adequate reliability and validity.

#### Social Self-Compassion Scale (SSCS)

This 12-item scale (Rose and Kocovski, 2021) measures perceived kindness and understanding toward the self after experiencing difficult social situations. The instructions are to rate each statement based on “How I typically act toward myself in difficult times.” Sample items include, “I try to be understanding and patient toward myself when I fall short of my social expectations” and “When I'm having a hard time in social situations, I give myself the caring and tenderness I need.” Items are rated on a scale ranging from 1 (*Almost Never*) to 5 (*Almost Always*). Higher scores indicate higher levels of social self-compassion. The psychometric properties of the SSCS are well-established across multiple studies with university students (Rose and Kocovski, 2021, 2025).

#### UCLA Loneliness Scale

This 20-item measure (Russell et al., 1980) assesses levels of perceived loneliness. Sample items include, “How often do you feel alone?” and “How often do you feel isolated from others?” Items are rated on a scale ranging from 1 (*Never*) to 4 (*Always*). Greater scores reflect a greater frequency of feeling alone. This scale possesses good psychometric properties (Russell et al., 1980).

#### COVID-19 state anxiety Sources Scale

This 12-item scale (Bareket-Bojmel et al., 2021) has four subscales comprising 3 items each, which assess COVID-19-related state anxiety in terms of health concerns, economic challenges, disruption to daily routine and change, and isolation. Health concerns are tapped by such items as, “I feel upset about the possibility the possibility of getting sick or that I already was sick with Corona.” Economic challenges are tapped by such items as, “I am worried about my economic situation.” Daily routines change is measured by items such as, “I feel upset by the fact that my daily routines have changed.” And finally, isolation is tapped by such items as, “I am worried about my loneliness.”

Items are rated on a scale ranging from 1 (*Not at all*) to 7 (*Very much*), with higher scores indicating higher levels of COVID-19 state anxiety. Research has shown that the subscales possess good internal consistency (Bareket-Bojmel et al., 2021).

#### Fear of COVID-19 Scale

This seven-item scale (Ahorsu et al., 2020) measures fear and anxiety about contracting COVID-19. Sample items include, “I am most afraid of Corona” and “I cannot sleep because I'm worrying about getting Corona.” Items are rated on a scale ranging from 1 (*Strongly Disagree*) to 5 (*Strongly Agree*), with greater scores reflecting a greater fear of COVID-19. This scale possesses good internal consistency, test-retest reliability, and concurrent validity (Ahorsu et al., 2020).

## Results

### Descriptive statistics

Table 1 presents the means, standard deviations, and alpha coefficients for all measures. All alpha coefficients were 0.83 or higher, exhibiting good internal consistency across all measures. The mean AMS score of 11.49 was slightly higher, but comparable to the mean for other university student samples (see Flett et al., 2023). The mean UCLA-20 score of 46.97 is considered to be within the moderate range for loneliness. Importantly, the mean SHQ score of 57.17 was considerably higher than the mean (SHQ = 51.2) found in a previous university sample (Heisel et al., 2003) who contrasted low suicidal ideators (mean SHQ = 47.8) with high suicidal ideators (mean SHQ = 62.6).

**Table 1:** Means, standard deviations, and alphas for all measures.

Variables	*M*	*SD*	Cronbach's alphas
Anti-Mattering	11.49	3.97	0.89
Hope	23.10	3.98	0.85
Hopelessness	5.56	5.08	0.90
Social Hopelessness	57.17	14.93	0.91
Social Self-Compassion	2.91	0.66	0.83
Loneliness	46.97	14.62	0.96
Total COVID Anxiety	42.38	16.21	0.91
Fear of COVID	16.59	6.19	0.89

N = 282 undergraduate students.

### Confirmatory factor analysis

A confirmatory factor analysis was performed using maximum likelihood estimation procedures to test for a one-factor solution with the five AMS items. We used the following fit indices to assess model fit: chi-square (χ^2^), comparative fit index (CFI), Tucker-Lewis index (TLI), standard root mean square residual (SRMR), and root mean square error of approximation (RMSEA). A model is considered to be an adequate fit if the CFA and TLI are greater than or equal to 0.90, and if the SRMR and RMSEA are less than or equal to 0.10 (Marsh et al., 2004). Additionally, the *p*_close_ is the *p*-value for a test of the null hypothesis that the RMSEA is less than or equal to 0.05, and a non-significant *p*_close_ indicates a close-fitting model. This model was an adequate fit, χ^2^_(5)_ = 17.190, *p* = 0.004, CFI = 0.984, TLI = 0.967, SRMR = 0.025, RMSEA = 0.093, 90% CI [0.047, 0.143], and *p*_close_ = 0.059. All AMS items had factor loadings of 0.77 or higher. Therefore, this analysis provides further support that the AMS is a unidimensional measure in this sample.

### Correlational analyses

Table 2 displays correlations among measures. Regarding feelings of not mattering, anti-mattering was positively correlated with hopelessness, social hopelessness, and loneliness. Anti-mattering was also negatively correlated with hope and social self-compassion. The highest of the correlations in Table 2 was between anti-mattering and loneliness. Supplementary analyses established that the robust correlation of 0.70 between anti-mattering and social hopelessness was significantly stronger than the correlation of 0.55 between anti-mattering and general hopelessness (*p* < 0.01).

**Table 2:** Correlations among anti-mattering, hope, hopelessness, social hopelessness, social self-compassion, loneliness, total COVID anxiety, and fear of COVID.

Measures	1	2	3	4	5	6	7	8
1. Anti-Mattering	–							
2. Hope	−0.46^**^	–						
3. Hopelessness	0.55^**^	−0.64^**^	–					
4. Social Hopelessness	0.70^**^	−0.49^**^	0.61^**^	–				
5. Social Self-Compassion	−0.52^**^	0.48^**^	−0.45^**^	−0.57^**^	–			
6. Loneliness	0.77^**^	−0.51^**^	0.55^**^	0.73^**^	−0.50^**^	–		
7. Total COVID Anxiety	0.41^**^	−0.27^**^	0.42^**^	0.46^**^	−0.32^**^	0.44^**^	–	
8. Fear of COVID	0.13^*^	−0.08	0.23^**^	0.17^**^	−0.07	0.14^*^	0.55^**^	–

*N* = 282. ^*^p < 0.05, ^**^p < 0.01, two-tailed.

In addition, anti-mattering was positively correlated with the total COVID anxiety score. While not included in the table, anti-mattering was positively correlated with all COVID state anxiety subscales: health (*r* = 0.19, *p* < 0.01), economic (*r* = 0.31, *p* < 0.001), routine (*r* = 0.22, *p* < 0.001), and isolation (*r* = 0.51, *p* < 0.001).

As can be seen in Table 2, hope was negatively correlated with hopelessness, social hopelessness, loneliness, total COVID anxiety, and positively correlated with social self-compassion. Hopelessness and social hopelessness were positively correlated with each other, as expected, and both indices of hopelessness were positively correlated with loneliness, total COVID anxiety, fear of COVID, and negatively correlated with social self-compassion. Moreover, social self-compassion was associated with greater levels of loneliness and total COVID anxiety. Loneliness was linked with greater total COVID anxiety and fear of COVID. Lastly, total COVID anxiety was positively correlated with fear of COVID.

### Regression analyses

Two multiple regression analyses were performed to examine which variables account for unique variance in loneliness and total COVID anxiety. The predictors in each instance were anti-mattering, social hopelessness, social self-compassion, and hope. Before conducting these analyses, we checked for normality of the outcome variables (i.e., loneliness and total COVID anxiety) and the distributions differed from normal. Thus, the robust bootstrapping procedure was employed because it does not impose the normality assumption. We generated 2000 bootstrap samples to provide estimates, standard errors, and 95% bias-corrected confidence intervals. Additionally, we checked the variance inflation factors for possible multicollinearity given some of the high correlations between predictors (anti-mattering, social hopelessness, and loneliness) and no signs of multicollinearity were found.

For the regression predicting loneliness, anti-mattering, social hopelessness, social self-compassion, and hope were all entered into the predictor block (see Table 3). This model significantly predicted 67.1% of the variance in loneliness scores, *F*_(4, 277)_ = 141.30, *p* < 0.001. Anti-mattering and social hopelessness were unique individual predictors of loneliness. In addition, hope was negatively associated with loneliness.

**Table 3:** Summary of multiple regressions for variables predicting loneliness and total COVID anxiety.

Variables	*R* ^2^	*B*	SE B	95% CI
Predicting loneliness				
Step 1	0.671^***^			
Anti-Mattering		1.74^***^	0.17	[1.40, 2.04]
Social Hopelessness		0.33^***^	0.05	[0.23, 0.44]
Social Self-Compassion		−0.12	1.06	[−2.25, 2.04]
Hope		−0.45^**^	0.15	[−0.73, −0.17]
Predicting total COVID anxiety				
Step 1	0.230^***^			
Anti-Mattering		0.67^*^	0.30	[0.11, 1.18]
Social Hopelessness		0.33^*^	0.09	[0.17, 0.51]
Social Self-Compassion		−1.44	1.72	[−4.70, 1.99]
Hope		−0.07	0.26	[−0.64, 0.43]

*N* = 282. ^*^p < 0.05, ^**^p < 0.01, and ^***^p < 0.001.

For the regression predicting total COVID anxiety, the same variables (i.e., anti-mattering, social hopelessness, social self-compassion, and hope) were again entered into the predictor block (see Table 3). This model significantly predicted 23% of the variance in total COVID anxiety scores, *F*_(4, 277)_ = 20.69, *p* < 0.001. Regarding individual predictors, anti-mattering and social hopelessness uniquely contributed to total COVID anxiety. In contrast to the regression analysis results for loneliness, hope was not a unique significant predictor in this analysis.

## Discussion

The period of pandemic restrictions provided an opportunity to observe psychological adaptation to isolation in emerging adults as if in a laboratory incubator. Indeed, our sample, as a whole, was found, perhaps most poignantly, to be experiencing moderate levels of loneliness. Previous reports described by Flett and Zangeneh (2020) highlighted the benefits of mattering during this period, that is, the sense of knowing that someone else cares (also see Matera et al., 2022). Importantly though, turning from a resilience to a vulnerability perspective, our previous research (McComb et al., 2020) with emerging adults during this period described what we termed the *double jeopardy* of feeling both lonely and insignificant, which was one of the first published empirical studies of the anti-mattering psychometric construct. The current study extends these resilience and vulnerability findings to a new understanding of links between anti-mattering and social self-compassion and social hopelessness as related to loneliness and anxious distress.

What we learned is that for the loneliest of emerging adults, they felt invisible (high levels of anti-mattering), unkind to themselves (low levels of social self-compassion), and hopeless and this low interpersonal hopelessness may reflect there is a lack of others in their lives who inspire or demonstrate caring. Our results indicated, as hypothesized, that anti-mattering, that is, the feeling of being insignificant to others, was associated with greater social hopelessness, greater hopelessness and reduced levels of hope. The associations between feelings of not mattering and hopelessness were exceptionally robust for social hopelessness and reduced sense of hope and suggested that the psychological distress and pain experienced by some students during pandemic isolation was accompanied by a bleak view of the future. Of crucial relevance to understanding what contributes to alienation was the finding of a significant correlation between anti-mattering and social hopelessness (*r* = 0.70), which suggests that anti-mattering is linked broadly with negative social expectancies. It is conceivable on the basis of our findings that students experiencing the pain of high levels of anti-mattering may also hold the belief that they will never matter to the people who are important to them (i.e., a social hopelessness about mattering). The negative correlation found between anti-mattering and dispositional hope extends the findings of an association between mattering and hope found by Somers et al. (2022) in their study of high school students. Even more so, based on regression analysis, it was anti-mattering and social hopelessness which were the strongest predictors of loneliness in our student sample, while hope was a negative predictor.

There are several implications that follow from the findings of anti-mattering, social hopelessness, and reduced hope as predictors of loneliness. In a previous *Frontiers in Psychology* special issue (Rokach and Goldberg, 2021), we discussed the double jeopardy of feelings of insignificance and being lonely (see McComb et al., 2020) and this association was also reflected in our current results. However, additionally and importantly, it now seems from the current results that these feelings of not mattering are also linked to the negative future component of the negative cognitive triad described by Beck (1967). The associations with social hopelessness and reduced hope are potentially alarming given the widely acknowledged role of hopelessness in suicide ideation and completed suicides (see Beck et al., 1975). While suicidality was not the focus of the present research, the results suggest it would be imperative to further investigate the role of feelings of not mattering in suicidality (e.g., Deas et al., 2023; Etherson et al., 2022) and more specifically levels of anti-mattering amongst those individuals who are seemingly at risk of suicide (Flett, 2024). The disturbingly high levels of social hopelessness in our sample approached the levels which have been found among individuals with suicidal ideation (Heisel et al., 2003). The very robust association of anti-mattering with social hopelessness may indicate that people who feel strongly that they do not matter to others may have a double jeopardy situation that is further amplified because their immediate painful lack of significance to others is projected into the future, perhaps even further fueling the self-perpetuating cycle of loneliness described by Rokach (2004). Taken together, among the loneliest, the potential for suicidality may perhaps be due, at least in part because of possible alienation, that they feel both invisible to others and are furthered pained by an absence of caring others available to inspire hope. The social hopelessness findings are most pertinent since young adults were found to be experiencing striking increases in suicidal ideation during this period of pandemic isolation (Liu et al., 2021). Again, however, future research would need to directly examine suicidality risk and levels of anti-mattering, particularly amongst young adults, as the present research did not specifically assess suicidality during or following the post-pandemic time period.

Other results attest to the relevance of the anti-mattering construct in terms of its associations with important positive psychology constructs. As expected, there was a strong negative link between anti-mattering and social self-compassion. This finding both replicates and extends previous research linking positive general feelings of mattering with elevated social self-compassion (see Rose and Kocovski, 2021). With regard to anti-mattering, people who feel like they are insignificant and feel like they go unheard by others seem to also have a hardened sense of self-compassion, that is, they tend to be unfairly self-critical and unkind to themselves.

As for the implications in terms of a person-centered perspective, Flett (2026) suggested that the pain of not mattering to others can become intolerable and grow into a form of what was described as unbearable insignificance. This is supported by the current results which suggest that certain students during the COVID-19 pandemic endured a toxic mixture consisting of anti-mattering, loneliness, social hopelessness, and a paucity of social self-compassion. It is difficult to imagine a positive outcome for anyone who is suffering through this combination for any substantial length of time. Much of this suffering is likely suffering in isolation with little contact or connection with others. The extent of their vulnerability was highlighted by the finding that anti-mattering was a key predictor of pandemic anxiety in the current study and was linked to all aspects of worries, namely health, economic, routine and isolation concerns. Such findings are consistent with the observation that the epidemic of loneliness seen during the pandemic was largely an epidemic of not mattering to others and feeling insignificant (Flett et al., 2025). As a real-life laboratory incubator, the pandemic circumstances made salient the psychological fragility of those people who feel particularly marginalized, who feel like they are expendable in the eyes of other people or if they are regarded at all, they are seen as a number rather than as a person (see Flett and Zangeneh, 2020). Results from the current study speak to the importance of addressing alienation among the most vulnerable through promoting and actually enacting more supportive communities, communities which build an individual's sense of community mattering (Flett, 2018). These supportive actions are not only relevant to the elderly, who understandably were a focus of identified concern during the pandemic, but also, based on the current study, speak to the need for efforts to reach out to vulnerable young adults. Having said that, emerging adults who feel invisible are also wary of others due to perceptions of stigma (see Pernice et al., 2017; Shannon et al., 2020) which can pose a further barrier to recovery of their sense of belongingness.

Parenthetically, although it was not our central focus, our results are also revealing in terms of the correlates of social self-compassion, a construct which is relatively new (Jiang et al., 2023) and has begun to be measured with the recently developed SSCS scale (Rose and Kocovski, 2021). Our results attested to the concurrent validity of this new measure in terms of the negative association between SSCS scores and anti-mattering, but we also showed that social self-compassion is linked with higher levels of hope and lower levels of social hopelessness, in keeping with this self-compassion construct seeming to be related to future expectancies. The strongest association with social self-compassion in our sample was its negative link with social hopelessness. The importance of promoting interpersonally-focused self-compassion is also suggested by the negative associations found between social self-compassion and both loneliness and pandemic anxiety, though the unique contributions of social self-compassion merit further investigation given that scores on this scale did not predict unique variance in loneliness or anxiety in this sample. Perhaps the lack of unique predictive significance is due, in part, to the strong negative link between social self-compassion and social hopelessness (*r* = −0.57); social hopelessness was a unique significant predictor in these regression analyses.

Given these observations, future research is needed to re-examine these associations from a longitudinal perspective. As well, in consideration of timeframe, the data were collected during the particularly unique social context of the pandemic and thus it is possible this may have influenced participants' self-reported experiences of loneliness. As such, it is important to bear in mind that loneliness levels during that time may be elevated due to contextual factors specific to the period of the data collection and thus some caution should be exercised when interpretating the magnitude and generalizability of the findings. However, there may very well be lingering effects of loneliness along with associated impacts that continue to be felt and experienced during the aftermath of the pandemic and future research could help to clarify the extent to which these research results have endured overtime. From a clinical perspective, the mental health ramifications of the social isolation experienced during the pandemic appear to continue reverberating into the present day.

It will also be important to examine the replicability of these results in future research in various samples, including participants from across the lifespan, but also clinical samples. Research is needed to examine mattering from a temporal perspective with a particular emphasis on whether current feelings of mattering are reflected in anticipated feelings of mattering or not mattering to others. Such research could further our understandings of the vicious cycles described by Rokach (2004) with regard to the future expectations of lonely people. Thoughts such as “I will never matter to those who matter to me” are likely frequent and intense. It would seem appropriate to develop a future outcomes anti-mattering measure that could serve as a supplement to the AMS, a self-report measure that would tap anticipated anti-mattering experiences. This proposed measure would have item content similar to the items found on the SHQ, but there would be a specific focus on future expectancies of being made to feel unimportant and irrelevant in general and more specific expectations (i.e., expecting to be treated as invisible and unheard).

In summary, the results of the current study further established the relevance of anti-mattering in terms of links with loneliness and elevated levels of anxiety among university students who were trying to contend with the altered life experiences of the pandemic, and now extends this previous research with potential implications most relevant to alienation. Our study showed uniquely that it was a sense of feeling like my voice goes unheard (elevated scores on the AMS), a sense that people do little to inspire hope in me (elevated scores on social hopelessness) and a reduced sense of hope which predicted loneliness during this time of pandemic isolation. Taken together, these findings describe a particular kind of vulnerability which seems relevant even today, that lonely young people who feel insignificant and who are failing to see hope for a better tomorrow, may be at heightened risk for suicidal ideation. However, as mentioned above, further research is needed to directly evaluate this association particularly with regards to risk of suicidality which was not directly assessed. We believe that the findings provide key messages for clinical and counseling interventions for lonely young adults, in that there should be a focus which addresses not only feelings of not mattering but also on their expectations of not mattering projected into the future. Moreover, given the salience of alienation suggested by the anti-mattering findings (which were perhaps made particularly relevant by the pandemic), there is a clear need to develop community-based proactive interventions that might impact feelings of personal insignificance. As well, the results suggest there may very well be a need to develop and engage in targeted initiatives and actions that reach out to young people. These initiatives can extend to those who are most vulnerable, with messages that incorporate inspiring reminders to be kind to themselves even amidst their loneliness, letting them know that there are others who not only recognize their struggles but also truly do care.

## Data Availability

The raw data supporting the conclusions of this article will be made available by the authors, without undue reservation.
